# Novel opportunities for treating complex neuropsychiatric and neurocognitive conditions based on recent developments with xanomeline

**DOI:** 10.3389/fpsyt.2025.1593341

**Published:** 2025-05-15

**Authors:** Valentin A. Pavlov

**Affiliations:** ^1^ The Feinstein Institutes for Medical Research, Northwell Health, Manhasset, NY, United States; ^2^ Department of Molecular Medicine, Zucker School of Medicine at Hofstra/Northwell, Hempstead, NY, United States; ^3^ Elmezzi Graduate School of Molecular Medicine, Manhasset, NY, United States

**Keywords:** schizophrenia, xanomeline, brain muscarinic receptors, Alzheimer’s disease, sepsis-associated encephalopathy, inflammation

## Introduction

Schizophrenia is a multifactorial chronic and frequently disabling mental disorder that reportedly affects about one percent of the world’s population (1, 2). Until recently, the pharmacological treatment of schizophrenia was almost entirely based on blocking brain dopamine D2 receptor signaling (2). While the approved therapeutics that act through this mechanism are efficacious in improving the positive symptoms in schizophrenia, they have considerable side effects including sedation, weight gain and motor impairment (2). They also have limited effectiveness in improving the negative and cognitive symptoms in patients with this disease (3). In addition to dopaminergic mechanisms, abundant experimental evidence reveals the important role of the brain cholinergic system and muscarinic acetylcholine receptors (mAChRs) as a therapeutic target in schizophrenia (4–6). Xanomeline is a centrally acting agonist of the M1 and M4 subtype of mAChRs with beneficial antipsychotic and cognitive effects (6, 7). Decades of research in preclinical and clinical settings and a series of recent clinical trials led to the FDA approval in September 2024 of xanomeline together with trospium for the treatment of schizophrenia (8). Trospium is a peripherally restricted nonspecific mAChR antagonist, which when given together with xanomeline mitigates the undesirable peripheral, mainly gastrointestinal effects of xanomeline. A recent review summarized the chronology and major findings from the clinical trials with xanomeline/trospium in schizophrenia (9). The approval of xanomeline/trospium is paradigm shifting, because for the first time in over 70 years a novel treatment which is not based on the mainstream concept of modulating D2 dopamine receptor signaling is available for schizophrenia. These recent developments may have broader implications: for reasons we summarize and discuss below, this drug combination can be explored in the future treatment of other complex neuropsychiatric and neurocognitive conditions such as Alzheimer’s disease (AD) and sepsis-associated encephalopathy (SAE) and long-term cognitive impairment.

## Xanomeline in the treatment of schizophrenia

There has been a substantial interest during the last few decades in studying the role of brain mAChRs in the pathogenesis of schizophrenia and targeting these receptors using pharmacological modalities for therapeutic benefit. Accumulated experimental evidence from preclinical and human brain imaging and post mortem studies was summarized in 2007 as a “muscarinic hypothesis of schizophrenia” (5). Studies in murine and non-human primate models have indicated the therapeutic potential of the centrally acting M1/M4 mAChR agonist xanomeline for treating psychosis and the mediating role of brain mAChRs (10–12). A small double-blind, placebo-controlled pilot study published in 2008, demonstrated the effectiveness of xanomeline in improving multiple symptom domains in patients with schizophrenia (13). However, side effects associated with xanomeline treatment, including gastrointestinal disturbances were also reported in this study. More recently, to mitigate these peripheral side effects in clinical studies with schizophrenia patients, xanomeline was combined with the peripherally acting mAChR antagonist trospium. A series of Phase 2 (14) and Phase 3 clinical trials (15, 16) demonstrated that treatment with xanomeline/trospium significantly decreased the positive and negative symptoms in people with schizophrenia who experience acute psychosis. These successful clinical trials led to the approval of xanomeline/trospium by the FDA for the treatment of adult patients with schizophrenia. Cognitive impairment, which may precede the onset of psychosis, is a core feature of schizophrenia (17). Importantly, xanomeline/trospium treatment significantly improved cognitive deficits in patients with acute schizophrenia who had preexisting cognitive deterioration as revealed by analysis of data pulled from the Phase 3 trials (18). These observations revealing the cognitive benefit of xanomeline/trospium among participants who had substantial impairment at baseline are consistent with the Phase 2 study data. Of note, as in the Phase 2 study, the beneficial cognitive effects of xanomeline/trospium were independent of changes in positive and negative symptoms, indicating that the cognitive improvements were not pseudo-specific (18).

## Xanomeline in the treatment of Alzheimer’s disease

The brain cholinergic system plays a key role in the regulation of attention, learning and memory (19). Characteristic neurodegeneration of brain (forebrain) neurons, with associated cognitive impairment is one of the cardinal features of AD, a debilitating and lethal brain neurodegenerative and neuropsychiatric disease and the most prevalent form of dementia (20, 21). In addition to schizophrenia, xanomeline has also been explored in the treatment of patients with AD. Almost 30 years ago (in 1997), in a large-scale multicenter, 6-month placebo-controlled clinical trial, xanomeline was found to significantly improve the cognitive function in AD patients (22). Intriguingly, xanomeline also improved psychosis in these patients (22). However, adverse, predominantly gastrointestinal events of xanomeline resulted in discontinued treatment of 52% of patients (22). Therefore, because of its peripheral side effects no further studies with xanomeline were performed despite its significant efficacy in counteracting the cognitive decline and neuropsychiatric symptoms in patients with AD.

## Brain functional impairment during sepsis

Sepsis is a multifactorial disorder that remains a number one killer in the intensive care units. According to its latest 2016 definition, sepsis is life-threatening organ dysfunction caused by a dysregulated host response to infection (23). One organ that is frequently, profoundly, and progressively affected during sepsis is the brain. The brain functional impairment and neuropsychiatric manifestations during sepsis, which include cognitive impairment and progression into delirium and coma, are characterized as sepsis-associated encephalopathy (SAE) (24–27). SAE can be defined as a diffuse cerebral dysfunction due to the dysregulated host response during sepsis secondary to infection which does not directly involve the central nervous system (24, 27). Brain alterations, including cognitive impairment and delirium occur very early during sepsis and are associated with increased hospital mortality (27–30). While the pathogenesis of SAE is not well understood, there is evidence from preclinical (31–33) and clinical studies for brain cholinergic alterations and hypofunction among the brain neurotransmitter changes that occur during sepsis (32, 34). As the brain cholinergic system plays a key role in the regulation of cognition, this cholinergic hypofunction may play a causative role in the severe cognitive impairment and delirium during sepsis (35).

Brain dysfunction and persistent cognitive impairment are also profound manifestations of sepsis long-term sequelae (36–38). Findings from a multicenter prospective cohort study revealed that one in four patients had cognitive impairment 12 months after critical illness comparable to that of mild AD (39). There is a correlation between SAE and especially delirium and the development of long-term cognitive dysfunction following hospital discharge (39, 40). Neuropsychiatric symptoms, including depression, anxiety, and post-traumatic stress disorder have also been documented in sepsis survivors (41, 42). In addition, an increased risk of suicide has been revealed in patients with prior hospitalization with infection, including sepsis (43). The long-term cognitive impairment and neuropsychiatric symptoms are key components of functional disability of sepsis survivors severely worsening their quality of life and associated with increased morbidity and mortality (38). While treating SAE encephalopathy and preventing and treating long-term cognitive impairment and neuropsychiatric manifestations in sepsis survivors are of fundamental value, there is a lack of targeted pharmacological modalities, and no specific guidelines have been currently implemented.

## Discussion of possibilities for using xanomeline/trospium in the treatment of AD and SAE

Psychosis and agitation are major and challenging to manage neuropsychiatric symptoms in AD (44, 45). Therefore, it is not surprising that based on accumulated evidence for its antipsychotic effects, clinical trials exploring the efficacy of xanomeline/trospium in treating AD psychosis are underway (46). Also, very recently, the emergence of psychosis in AD was correlated with increased plasma levels of p-tau phosphorylated at threonine 181 (p-tau181) and neurofilament light chain protein (NfL) and their utility as biomarkers of neuropsychiatric illness in AD was suggested (47). Therefore, it would be intriguing to determine whether plasma p-tau181 and NfL levels are altered in AD patients treated with xanomeline/trospium and corelate with antipsychotic drug effects in these trials.

The effects of xanomeline/trospium in AD might be far reaching and not just restricted to treating psychosis. This speculation is strongly supported by the prior study which showed that xanomeline monotherapy significantly improved cognitive domains in AD patients (22). It appears that the main obstacle for its use, i.e., severe peripheral side effects that for a long-time hampered progress, can now be addressed by including trospium. Hence, evaluating the effects of xanomeline on cognitive function in patients with AD, utilizing the double-blind placebo-controlled design in the 1997 study but with the addition of trospium is warranted. The use of this therapy can also be considered for treating cognitive impairment in the context of other disorders.

As previously noted, xanomeline is an M1/M4 m AChR agonist. It is not clear whether xanomeline beneficial effects in schizophrenia are due to the action on the brain M1 or the M4 subtype or both. The brain M1 and M4 mAChRs have different synaptic location and different function (48–50). The M1 subtype is predominantly postsynaptic and plays a major role in processing cholinergic neurotransmission in the cortex and the hippocampus with an essential function in the regulation of cognition (48). Of note, studies with mice lacking specific mAChR subtypes have shown no direct involvement of the M1 subtype in the control of brain (striatal) dopamine release (51). The brain M4 subtype is predominantly presynaptic and associated with modulation of neurotransmitter, including dopaminergic system (50–53). There is some evidence linking the antipsychotic-like effects of xanomeline and its efficacy in treating positive and negative symptoms in patients with schizophrenia to its action on the M4 subtype, while the cognitive effects might be mainly associated with activation of M1 mAChR signaling (6, 54).

The postsynaptically located M1 mAChR is largely preserved during the neurodegenerative alterations in AD (21, 55, 56). Thus, the M1 mAChR presents a viable target for therapeutic strategies aimed at counteracting cognitive impairment in AD by activating M1 mAChR signaling (21, 48, 57, 58). Preclinical studies have also demonstrated a role for activation of M1 mAChR signaling in reducing other characteristic features of AD pathology, including amyloid plaques, mainly composed of the amyloid-β peptide and neurofibrillary tangles, comprised of hyperphosphorylated aggregates of the tau protein (59, 60). These findings have a provided a rationale for proposing the use of M1 mAChR agonists in potentially disease-modifying therapies for AD (57, 58). Therefore, xanomeline acting on the brain M1 mAChR may have additional beneficial effects in AD.

In addition to regulation of cognition, brain (forebrain) mAChR and specifically the M1 mAChR are implicated in the regulation of peripheral cytokine levels and inflammation (61–66). Administration of xanomeline or BQCA, a selective positive allosteric modulator of the M1 mAChR with demonstrated pro-cognitive effects (67) significantly suppresses circulating pro-inflammatory cytokine levels in mice with endotoxemia (62, 65). Xanomeline reduces serum pro-inflammatory cytokine levels, ameliorates sickness behavior, and improves survival in endotoxemia and in a preclinical mouse model of sepsis (62, 68). The anti-inflammatory effects of xanomeline are mediated through brain mAChRs, because they are inhibited in mice with pharmacological blockade of these receptors (62). These effects also require signaling through the vagus nerve (62). The vagus nerve is a key constituent of a major physiological mechanism termed *the inflammatory reflex* that links the brain and the immune system and controls peripheral cytokine responses (62, 69, 70).

Accumulated experimental evidence during the last 20 years has revealed a role for systemic inflammation in the pathogenesis of schizophrenia and as a new therapeutic target in the treatment of this disease (7, 71). As we have recently proposed, the anti-inflammatory activity of xanomeline may contribute to its remarkable efficacy in alleviating multiple symptoms in patients with schizophrenia (7). Such a possibility can be evaluated in future clinical studies in which subclusters of patients with increased inflammation can be reliably identified using machine learning, and additional analysis of xanomeline anti-inflammatory effects in this subset of patients is performed (7).

There is also evidence for systemic inflammation that is linked with brain inflammation in the pathophysiology of AD (72–74). It has also been reported that both acute and chronic inflammation and increased levels of pro-inflammatory cytokines are associated with exacerbated cognitive decline in patients with AD (75). Therefore, it is intriguing to consider that the beneficial effects of xanomeline on cognition in patients with AD will be amplified by its anti-inflammatory effects. Such a possibility can be experimentally and more specifically tested in AD patients with increased peripheral cytokine levels by implementing the same approach as proposed for patients with schizophrenia.

Increased circulating pro-inflammatory cytokine levels and systemic inflammation linked to brain inflammation (neuroinflammation) and brain cholinergic hypofunction have been implicated in SAE (25, 32, 76, 77). Of note, administration of xanomeline in a murine model of sepsis improves survival and ameliorates markers of immune dysregulation and inflammation (62, 68). There is also evidence for immune dysregulation and increased levels of pro-inflammatory cytokines and inflammation in sepsis survivors (31, 78, 79). Long-term cognitive impairment with symptoms that are similar to those in patients with mild AD has also been reported in sepsis survivors (38, 39). Importantly, for most patients, this profound cognitive deterioration in sepsis survivors was not related to cognitive impairment before their admission to the intensive care unit (39). It was also documented in both old and young patients, regardless of the burden of coexisting conditions at baseline (39). While of primary clinical significance, treating SAE is challenging as no targeted treatments are currently available. The anti-inflammatory and beneficial cognitive efficacy of xanomeline (together with trospium) can be explored in new therapies for SAE and the long-term cognitive impairment in sepsis survivors.

In conclusion, based on evidence and reasoning we outline here, xanomeline/trospium - a therapeutic modality that was very recently approved for schizophrenia - can also be considered in novel therapeutic strategies for AD and SAE and long-term cognitive deficits.
